# The impact of maternal systemic diseases on the occurrence of cleft lip and palate in newborns: a narrative review

**DOI:** 10.3389/fpubh.2025.1568140

**Published:** 2025-08-01

**Authors:** Hao Sui, Meijun Du, Jiali Chen, Renjie Yang, Bing Shi, Hanyao Huang, Yan Wang

**Affiliations:** ^1^State Key Laboratory of Oral Diseases and National Clinical Research Center for Oral Diseases, Department of Oral and Maxillofacial Surgery, West China Hospital of Stomatology, Sichuan University, Chengdu, Sichuan, China; ^2^State Key Laboratory of Oral Diseases and National Clinical Research Center for Oral Diseases and Eastern Clinic, West China Hospital of Stomatology, Sichuan University, Chengdu, Sichuan, China

**Keywords:** CLP, maternal, pregnancy, systemic diseases, risk factors

## Abstract

Cleft lip and palate (CLP) is a prevalent congenital anomaly of the maxillofacial region, characterized by abnormal openings in the lip or palate. This condition, affecting approximately 1 in 700 newborns globally, can manifest as cleft lip only, cleft palate only, or both. The etiology of CLP remains multifactorial, involving genetic and environmental influences, with maternal systemic diseases during pregnancy emerging as significant risk factors. Conditions such as circulatory disorders, endocrine and metabolic disorders, infectious diseases, and autoimmune diseases have been associated with increased CLP incidence. These maternal health issues can disrupt normal embryonic development, leading to cleft formation and affecting the child’s overall wellbeing, including feeding, speech, dental health, and psychological state. This review explores the relationship between maternal systemic diseases, including circulatory, endocrine and metabolic, infectious, and autoimmune disorders, and the occurrence of CLP in newborns. Understanding these connections is crucial for improving maternal health during pregnancy and reducing the risk of CLP, highlighting the importance of early monitoring and intervention.

## 1 Introduction

CLP is a common congenital defect of the maxillofacial region and is the most common craniofacial malformation in humans. The global average incidence rate among newborns is 1/700 (1). It is characterized by an abnormal opening or gap in the lip or the upper palate. Depending on the area involved, it can be classified into three types: cleft lip only, cleft palate only, and CLP (1). CLP can occur independently, which is more common and referred to as non-syndromic CLP (2). It can also occur in conjunction with other congenital diseases, known as syndromic CLP, with congenital heart disease being the most common associated condition (3). From an embryonic development perspective, during the sixth week of normal embryonic development, the upper lip forms as the globular process fuses with the maxillary processes on both sides (4). In this process, the globular process grows toward the oral cavity, forming the primary palate, and by the end of the seventh week, the lateral palatine processes, which develop from the maxillary processes, fuse to form the upper palate (4, 5). Any disruption in the fusion process of the globular process and the palate during embryonic development can lead to the occurrence of cleft lip, cleft palate, or CLP (6). CLP not only affect aesthetics and oral function but also result in related complications such as feeding difficulties, speech problems, dental defects, malocclusion, abnormal facial growth, and middle ear infections (7). These issues can lead to lifelong psychosocial problems, significantly impacting the mental health of the affected child (8).

The exact cause of CLP is not fully understood. It is currently believed to result from a combination of genetic and environmental factors (9). Genetic mutations or variations, along with parental lifestyle and living environment, such as excessive alcohol consumption, smoking, inadequate intake of vitamins or minerals during pregnancy, and the use of certain medications during pregnancy (such as painkillers, antibiotics, and antihypertensive drugs), can all increase the likelihood of a newborn developing CLP (10). Additionally, factors such as parental consanguinity, educational level, and health status may also be associated with the occurrence of CLP in children (1, 9, 11–14).

In recent years, an increasing number of studies have begun focusing on the relationship between maternal health conditions and the occurrence of CLP in newborns. Maternal systemic diseases may negatively impact pregnancy outcomes. Conditions such as cardiovascular diseases (e.g., hypertension, atherosclerosis), diabetes, obesity, and autoimmune diseases (e.g., lupus) have been shown to increase the risk of complications during pregnancy (15–20). Metabolic disorders during pregnancy can also raise the risk of adverse outcomes and significantly affect fetal development, including the formation and fusion of oral and facial structures (21, 22). Therefore, maternal systemic diseases during fetal development may be closely related to the occurrence of CLP in newborns, increasing the risk of this congenital condition (16, 23, 24). This review will explore the relationship between maternal systemic diseases and the occurrence of congenital CLP in children, focusing on conditions such as circulatory system diseases, endocrine and metabolic disorders, infectious diseases, and autoimmune diseases (Table 1). The aim is to review and synthesize available evidence linking maternal systemic diseases with CLP in newborns, to highlight areas where further basic and clinical research are needed.

**Table 1:** The relationship between systemic diseases in pregnant women and congenital malformations, and relationship between drug exposure and congenital malformations.

The relationship between systemic diseases in pregnant women and congenital malformations				
Category	Description	Findings	Study types	References
Circulatory disorders	Disorders such as gestational hypertension, pre-eclampsia, and heart disease.	Gestational hypertension and pre-eclampsia increase the risk of cleft lip and palate, especially when pre-eclampsia is superimposed on pre-existing hypertension. Increased oxidative stress may be a contributing factor.	Population-based cohort studies, case–control studies, systematic review and experimental animal studies.	(31) (32) (33) (34) (35)
Endocrine metabolic system diseases	Includes gestational diabetes and obesity.	Gestational diabetes and maternal obesity are associated with a higher risk of cleft lip and palate, due to inflammation, oxidative stress, and changes in fetal maxillofacial development.	Cross-sectional and population-based studies, cohort studies, case–control studies, meta-analyses, and experimental animal studies.	(40) (38) (39) (37) (36) (43) (42) (41) (45) (46) (47) (36) (48) (49) (53) (52) (50) (45) (51)
Infectious diseases	Acute infections (e.g., influenza) and chronic (e.g., periodontitis).	Infections and inflammatory diseases during pregnancy can increase the risk of cleft lip and palate due to heightened inflammatory responses.	Nested case–control study, prospective cohort study, and experimental animal studies.	(54) (55) (56) (57) (58) (59) (60) (62) (63) (64) (65) (15) (66)
Autoimmune diseases	Includes rheumatoid arthritis, SLE, IBD, and MS.	Autoimmune diseases may lead to an increased risk of cleft lip and palate, likely due to increased inflammatory markers and medication effects.	Population-based case–control study, systematic review, meta-analysis, and experimental animal study.	(67) (68) (69) (70) (71) (72) (73) (74) (75)

## 2 Methods

### 2.1 Search strategy

This study was conducted as a narrative review of the literature focusing on the association between maternal systemic diseases and orofacial clefts in newborns. Relevant articles were identified by searching PubMed, Web of Science, and Embase databases for studies published in English from 2000 to 2024.

Search strategy utilizes the following key terms: Maternal Condition Terms: Pregnancy-related: “pregnant women, ““maternal, ““pregnancy complications”; Circulatory: “hypertension, ““preeclampsia, ““hypertensive disorders of pregnancy”; Metabolic: “diabetes mellitus, ““gestational diabetes, ““obesity, ““maternal obesity, ““metabolic syndrome”; Infectious: “infection, ““viral infection, ““bacterial infection, ““periodontal disease, ““periodontitis, ““fever”; Autoimmune: “autoimmune disease, ““lupus, ““systemic lupus erythematosus, ““inflammatory bowel disease”; Medication exposure: “antiepileptic drugs, ““anticonvulsants, ““beta-blockers,” “angiotensin converting enzyme inhibitors,” “antibiotics,” “corticosteroids.” Outcome Terms: “cleft lip,” “cleft palate,” “orofacial cleft,” “cleft,” “congenital anomaly,” “congenital malformation,” “birth defect.”

Titles and abstracts identified through the search were independently screened by two reviewers for relevance. Full-text articles were then assessed by eligibility criteria. Disagreements regarding article inclusion were resolved by discussion or consultation with a third reviewer.

### 2.2 Eligibility criteria

We specifically included case–control studies, prospective and retrospective cohort studies, comprehensive cross-sectional analyses, and methodologically sound meta-analyses that provided quantitative assessments of these relationships.

We excluded studies focused exclusively on genetic factors without consideration of maternal systemic conditions. Similarly, animal studies lacking human translational components were omitted from the primary analysis. Case reports and small case series with insufficient statistical power were also excluded from our main analysis, though they were occasionally referenced to provide mechanistic insights or to highlight potential pathophysiological pathways warranting further investigation.

## 3 Systemic diseases and their impact

Systemic diseases encompass a broad spectrum of disorders that affect multiple organs and body systems simultaneously. These conditions can be classified into several major categories, including circulatory disorders, endocrine and metabolic disorders, infectious diseases, and autoimmune diseases. Research has shown that inflammation plays a fundamental role in the etiology of these diseases across the life span (25). The inflammatory response to cellular injury or pathogen-associated signals—particularly involving damage-associated molecular patterns (DAMPs) such as high-mobility group box 1 (HMGB1), S100 proteins, heat shock proteins (HSPs), and nucleic acids—serves as both an initiating factor and a perpetuating mechanism in disease progression (26, 27). Immune system dysregulation represents a central feature in systemic diseases, manifesting through various mechanisms, including altered immune cell function and inflammatory mediator production (28). This dysregulation can lead to a state of chronic systemic inflammation, which has been linked to multiple pathological conditions and organ dysfunction (29, 30).

### 3.1 Circulatory disorders

Circulatory disorders during pregnancy encompass heart disease, gestational hypertension, and pre-eclampsia (31). Although heart disease in pregnancy is considered a circulatory disorder, current evidence does not support a direct association between maternal heart disease and non-syndromic CLP. But it has been reported that hypertension in pregnancy and pre-eclampsia are associated with the development of non-syndromic CLP (32). A cohort study comprising 2.49 million newborns demonstrated that gestational hypertension and pre-eclampsia elevated the likelihood of non-syndromic CLP in newborns (33). Furthermore, the risk of having a child with non-syndromic CLP in a woman with pre-eclampsia superimposed on pre-existing hypertension was more than twice as high as the risk in a woman without hypertension (33). A further cross-sectional study, based on data from 29 countries, demonstrated that chronic maternal hypertension was associated with an increase in the prevalence of CLP of more than four-fold, with an eight-fold increase observed in cases where the mother had pre-eclampsia (32). The levels of circulating oxidative stress markers, such as malondialdehyde (MDA) and superoxide dismutase, were significantly elevated in mothers with gestational hypertension or pre-eclampsia compared to those with a normal pregnancy (34). Furthermore, MDA levels correlated with the severity of pre-eclampsia, suggesting that gestational hypertension and pre-eclampsia place mothers in a state of oxidative stress imbalance (35); this imbalance may be a probable cause of CLP in the fetus.

### 3.2 Endocrine and metabolic disorders

Disorders of the endocrine and metabolic system that predispose mothers during pregnancy include gestational diabetes and gestational obesity (36–40). Gestational diabetes mellitus (GDM), defined as abnormal glucose tolerance initiated or first detected during pregnancy, has been demonstrated to significantly affect fetal development, with an increased risk of developing CLP (37–40). The hyperglycemic environment of pregnancy in diabetic rats has been demonstrated to result in alterations in inositol and prostaglandin metabolism and increased levels of reactive oxygen species (ROS), which in turn affects the development of neural crest-derived organs, leading to a variety of craniofacial malformations, including CLP (41–44).

In addition, GDM has been shown to induce a chronic inflammatory state in the mother (45). Women with GDM have been observed to exhibit elevated levels of several blood inflammatory markers, including the neutrophil-to-lymphocyte ratio (NLR), the platelet-to-lymphocyte ratio (PLR), the white blood cell (WBC) count, and the neutrophil count, in comparison to healthy pregnant women (46). Plasma protein profiling has revealed the presence of a heightened abundance of pro-inflammatory proteins, which include pigment epithelium-derived factor (PEDF), proteoglycan (PRG4), and fibronectin 1 (FN1), in the plasma of women prior to the diagnosis of gestational diabetes, suggesting that the inflammatory state was present long before the diagnosis of diabetes mellitus (47). It may affect the maxillofacial development of the fetus from early pregnancy onwards.

Maternal obesity in the early stages of pregnancy is positively correlated with the risk of CLP in the offspring (36), and the incidence of CLP is positively correlated with maternal obesity (37). Maternal obesity is associated with chronic metabolic inflammation (48); maternal obesity prior to pregnancy results in the accumulation of macrophages in the placenta (49), which in turn leads to an increase in the levels of reactive oxygen species and pro-inflammatory cytokines in maternal plasma, including IL-8, IL-6, CRP, TNF-*α* and IFN-*γ* (45, 50–53), which in turn affects fetal maxillofacial development, leading to the development of CLP.

### 3.3 Infectious diseases

Common infectious diseases in pregnant mothers include a variety of acute infections and chronic inflammatory diseases (54). Acute infections such as influenza, acute bronchitis, and urinary tract infections have been shown to be associated with the risk of developing CLP (55). These acute infections typically present with sudden onset of symptoms and can cause maternal fever, which is particularly concerning during early pregnancy. Research has shown that maternal fever above 38.5°C during the first trimester significantly increases the risk of orofacial clefts (56). Studies showed that untreated maternal fever was associated with a higher risk of oral clefts, particularly non-isolated clefts, although the risk did not significantly differ by fever severity (57). These acute infections can affect fetal development through various mechanisms, including direct pathogen effects and maternal inflammatory responses (58).

Chronic maternal infections have been identified as significant risk factors for orofacial clefts. Common chronic infections during pregnancy include periodontitis, chronic cytomegalovirus (CMV) and hepatitis B virus (HBV) infection. Among these, periodontitis is characterized by periodontal tissue inflammation caused by gram-negative, anaerobic bacteria in the subgingival region (59). These pathogenic microorganisms trigger systemic inflammatory responses, elevating pro-inflammatory cytokines including IL-1, TNF-*α*, IL-6, and prostaglandin E2 (60–62). Similarly, maternal CMV infection has been associated with increased inflammatory markers and adverse fetal outcomes (63). Chronic HBV infection during pregnancy can lead to persistent inflammation and elevated cytokine levels (64). These chronic infections share common pathogenic mechanisms whereby inflammatory mediators can enter systemic circulation, potentially crossing the placental barrier and interfering with normal craniofacial development during the critical embryonic period (15, 65, 66).

### 3.4 Autoimmune diseases

Autoimmune diseases are characterized by a female predominance and first manifest during the reproductive phase (67). Autoimmune diseases such as rheumatoid arthritis, systemic lupus erythematosus (SLE), inflammatory bowel disease (IBD), and multiple sclerosis (MS) are common autoimmune diseases during pregnancy, and their prevalence has increased in recent years (68, 69). Changes in physiology and hormone levels associated with pregnancy can lead to SLE flares and an increased inflammatory response in the body (70). Mothers with SLE have elevated serum levels of the pro-inflammatory cytokine IL-10, accompanied by elevated levels of several chemokines (71). Although it has been reported that mothers with SLE have a higher risk of giving birth to a child with CLP, the reason for this is unclear and may be related to the use of glucocorticoids (72).

IBD, comprising Crohn’s disease and ulcerative colitis, is a chronic immune-mediated disorder frequently diagnosed during reproductive years (73). The disease is characterized by dysregulated immune responses and elevated pro-inflammatory cytokines, particularly TNF-*α*, IL-1β, and IL-6, which contribute to persistent intestinal inflammation (74). Studies have shown that patients with active IBD have a significantly higher risk of adverse pregnancy outcomes, including preterm birth, spontaneous abortion, and infants small for gestational age (75). However, the specific relationship between maternal IBD and the development of CLP requires further investigation, as current evidence is limited, and mechanisms remain unclear.

### 3.5 Intrauterine exposure to drugs

During pregnancy, women with systemic diseases often need medication to manage their conditions and prevent acute flare-ups. It is important to recognize that the potential teratogenic effects of these drugs may also contribute to the overall risk of congenital anomalies in the offspring. Despite the presence of the placental barrier, nearly all drugs taken by pregnant women can enter the fetal circulation to some extent through passive diffusion (76). Some medications used to treat systemic illnesses are linked to an increased risk of birth defects such as CLP (77). For example, beta blockers and angiotensin converting enzyme inhibitors (ACEI) for hypertension (78, 79), antibiotics for infections (80, 81), and immunosuppressant drugs for autoimmune disorders have been associated with these outcomes (82–84). Antiepileptic drugs pose a high risk of birth defects, with valproate being associated with a 10.3% incidence of severe congenital malformations (85) (Table 2). Therefore, it is crucial to carefully weigh the benefits of controlling systemic conditions during pregnancy against the potential risks these medications pose to the fetus. Given that many drugs have dose-dependent teratogenic effects (86), it is advisable to use the lowest effective doses when treating systemic diseases in pregnant women.

**Table 2:** The relationship between drug exposure and congenital malformations.

Category	Drugs	Findings	References
Circulatory disorders	Beta blockers	Exposure to beta-blockers during the first trimester of pregnancy is associated with a 3 in 1,000 likelihood of cleft lip or palate.	(78)
	ACEI	Maternal use of ACE inhibitors in the first trimester has a risk profile similar to the use of other antihypertensives regarding malformations in live born offspring.	(79)
Infectious diseases	Antibiotics	The use of nitrofurantoin during pregnancy has been associated with an increased risk of cleft lip with or without cleft palate, with an adjusted odds ratio (AOR) of 2.1 (95% CI: 1.2–3.9).	(81) (80)
Autoimmune diseases	Mycophenolate mofetil	Exposure to mycophenolate mofetil in early pregnancy causes a variety of road deformities including cleft lip and palate.	(84) (82) (83)
	Corticosteroids	There is a moderately increased risk of CLEFT LIP AND PALATE among women who use corticosteroids during early pregnancy. Exposure to corticosteroids in the first trimester increased the risk of cleft lip and palate in newborns by 1.2%。	
Others	Antiepileptic drugs	Valproate being associated with a 10.3% incidence of severe congenital malformations	(85)

## 4 Conclusion

CLP are relatively common birth defects, with varying prevalence rates observed in different populations (1). The impact of CLP on an individual may vary depending on the severity and extent of the condition, affecting the overall quality of life and emotional wellbeing of the affected child, as well as their appearance, feeding, articulation, dentition, hearing, and psychosocial wellbeing. The treatment of CLP requires a multidisciplinary approach, involving a sequence of timely and age-appropriate interventions. These include surgeries for the lip and palate, followed by postoperative orthodontics, orthognathic surgery, and essential psychological support to ensure the patient’s overall physiological and psychological wellbeing (9). Nevertheless, further investigation is required to elucidate the impact of maternal health on the occurrence of CLP.

**Figure 1:**
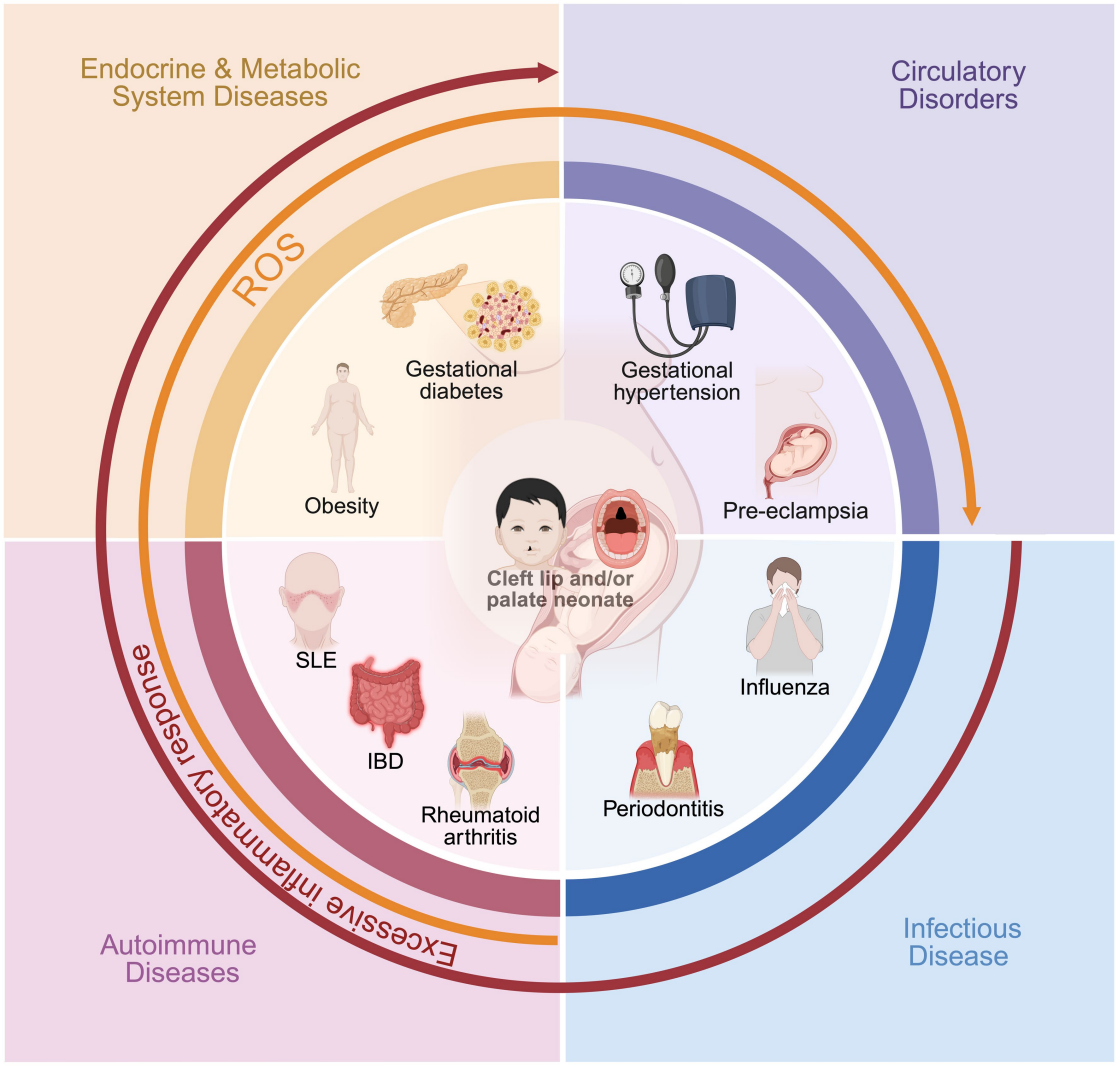
The impact of maternal systemic diseases on the occurrence of cleft lip and palate in newborns.

This review aims to introduce the relationship between maternal circulatory, endocrine and metabolic, infectious, and autoimmune diseases during pregnancy and the development of CLP in newborns (Figure 1). The presence of the disease before or during mid-pregnancy may elevate the risk of fetal birth defects (87). Large-scale cohort studies have shown that gestational hypertension and pre-eclampsia notably increase the risk of non-syndromic CLP, possibly through oxidative stress and impaired placental function, highlighting the importance of early detection and blood pressure management in clinical practice. In terms of metabolic factors, conditions such as gestational diabetes mellitus and maternal obesity contribute to CLP risk by inducing chronic inflammatory processes and disrupting critical signaling pathways in neural crest development; recent research points to a continuum of risk that scales with the severity of these metabolic disturbances. For infectious diseases, the presence of acute maternal fever or persistent infections—such as influenza, hepatitis B, or periodontitis—during early pregnancy significantly raises the likelihood of CLP, likely via direct teratogenicity and heightened systemic inflammation, though more mechanistic studies and randomized trials are needed. The influence of autoimmune diseases (like SLE and IBD) is increasingly recognized, with both disease activity and treatment playing roles in mediating risk, yet the distinction between drug effect and underlying immune dysfunction requires further study. Medication exposures, especially certain antihypertensives and antiepileptics, also warrant individualized risk–benefit analysis, as both drug type and timing can influence CLP occurrence. Collectively, these findings underscore the necessity of comprehensive, condition-specific research to clarify the molecular mechanisms and gene–environment interactions underlying each systemic disease. Additionally, many other factors increase the risk of a newborn having a CLP, such as high maternal exposure to alcohol during pregnancy, smoking, stress during pregnancy, and the use of assisted reproduction techniques, all of which are significantly associated with the birth of a child with a CLP (37, 88–90).

By comprehending the detrimental effects of CLP and employing preventative measures, it is conceivable that the prevalence of this condition could be diminished, consequently enhancing the overall quality of life for those affected (91, 92). Furthermore, routine antenatal assessments and the prompt identification of potential issues can facilitate the management and planning of CLP treatment (93). Therefore, it is recommended to emphasize the need for early pregnancy follow-up in women with systemic diseases, receive appropriate medical care and close monitoring during pregnancy to control identified risk factors and receive appropriate protective measures (92), in order to reduce the chances of neonates developing CLP occurrence and to ensure the best possible pregnancy outcome for both mother and baby.
